# Detrimental effect of increased blood pressure variability on clinical outcome in acute ischemic stroke treated with reperfusion therapy: a case control study

**DOI:** 10.1186/s12883-022-02605-5

**Published:** 2022-03-12

**Authors:** Jingcui Qin, Qing He, Zhijun Zhang

**Affiliations:** 1 Department of Neurology, School of Medicine, The Key Laboratory of Developmental Genes and Human Disease, Affiliated ZhongDa Hospital, Neuropsychiatric Institute, Southeast University, 87 Dingjiaqiao Road, Nanjing, 210009 Jiangsu China; 2 Department of Neurology, Xuzhou First People’s Hospital, The Affiliated Hospital of China University of Mining and Technology, The Affiliated Xuzhou Municipal Hospital of Xuzhou Medical University, Xuzhou, China

**Keywords:** Acute ischemic stroke, Blood pressure variability, Clinical outcome, Reperfusion therapy

## Abstract

**Background:**

Blood pressure variability (BPV) is related to clinical outcome in acute ischemic stroke (AIS) treated with reperfusion therapy, but still is not included in clinical practice. This study aims to associate BPV during the first week of AIS with the outcome at three months.

**Methods:**

We included 236 AIS patients treated with reperfusion therapy, and then divided them into good outcome and poor outcome groups. BPV measurements, including standard deviation, coefficient of variation, average real variability (ARV), and range, were recorded at stages of 2 h, 24 h, and 7 days after reperfusion therapy, respectively. These measurements were compared between the two groups. Then, ROC curve analysis examined the ability of BPV measurements in differentiating good and poor outcome groups; logistic regression analysis detected variables associated with clinical outcome in all subjects.

**Results:**

The good outcome group exhibited significantly less BPV at all stages relative to the poor outcome group. The BPV measurements exhibited the potential to differentiate the two groups by the ROC curve analysis, especially for those at the 24-h stage. Higher ARV of diastolic BP and higher range of systolic BP at the 24-h stage, together with greater disease severity at baseline, were associated with poor clinical outcome.

**Conclusions:**

Greater BPV during the first week of AIS is associated with poor outcome for the patients treated with reperfusion therapy. The BPV measurements play an important role in modulating clinical outcome, and have the potential to be included in future AIS guidelines.

## Background

Timely reperfusion therapy, including intravenous thrombolysis (IVT) and endovascular thrombectomy (EVT), is strongly recommended in the acute phase of acute ischemic stroke (AIS) [1]. Clinical outcome after reperfusion therapy is determined by various factors such as age, time to and success of recanalization, baseline stroke severity, and arterial blood pressure (BP). Among these factors, BP is an important modifiable factor in AIS [2]. Current guidelines on AIS recommend absolute BP level below a certain level during and after reperfusion therapy to keep patients away from symptomatic intracerebral hemorrhage [1, 3]. Recent studies proposed a U-shape relationship between absolute BP level and clinical outcome, which extreme high and low BP level are associated with poor outcomes [4]. However, intensive BP lowing to around 140 mmHg did not benefit in better clinical outcome relative to guideline treatment [5]. Therefore, absolute BP level alone is insufficient to affect clinical outcome for such patients.

Recently, neurologists propose that BP variability (BPV) may also play a role in affecting clinical outcome for AIS patients, in addition to the absolute BP level [6]. It is expected that the ischemic tissue would be more susceptible to blood pressure fluctuation given the impairment of cerebral autoregulation by stroke [7]. Increased BPV is linked to infarct growth and higher rates of mortality and disability [8, 9]. Similarly, for AIS patients treated with reperfusion therapy, prevalent studies indicate the association between increased BPV within 24-48 h after stroke onset and worse clinical outcome at three months [9–11]. By contrast, such an association did not emerge in a study investigating AIS patients receiving EVT [12]. Different measurements of BPV and different time points at which data acquired among studies would impede a recommendation to clinical practice in guidelines.

Currently, the most common BPV measurements include standard deviation (SD), coefficient of variation (CV), average real variability (ARV), and range (R). Although most of the studies demonstrate the detrimental effect of increased BPV during the first 24-48 h after reperfusion therapy, it is largely unknown whether such an effect extends to the first week of the stroke onset. In addition, to promote BPV measurements into clinical practice, it is necessary to evaluate the extent to which the BPV measurements are associated with clinical outcome among various clinical variables. Therefore, this study included a cohort of AIS patients treated with reperfusion therapy, and then divided them into good outcome and poor outcome groups according to the Modified Rankin Scale (mRS) at day 90 after AIS. BPV measurements and other clinical variables were compared between the two groups. A receiver operating characteristic (ROC) curve analysis was used to examine whether and how BPV measurements differentiate AIS patients with different clinical outcomes. In addition, a logistic regression analysis was used to evaluate the role of BPV in affecting clinical outcome among various clinical variables. In all, this study evaluated the association of BPV during the first week after reperfusion therapy with clinical outcome in AIS. The results provide empirical evidence for managing BPV in acute phase of AIS in clinical practice.

## Methods

### Subjects

All subjects were recruited from the Xuzhou first people's Hospital. The study was approved by The Ethics Committee of Xuzhou first people's Hospital and conducted according to the Declaration of Helsinki. Written informed consents were obtained individually. This study included 236 AIS patients presenting within 4.5 h after the symptom onset. The group of the 174 subjects whose 90-day mRS score was less than two was defined as the good outcome group, whereas the group of the remaining 62 subjects whose 90-day mRS score was higher than or equal to 2 was defined as the poor outcome group.

### Inclusion and exclusion criteria

Each subject met the following inclusion criteria: 1) they were treated with reperfusion therapy, including IVT, EVT, or bridging therapy; 2) they underwent multi-modal computerized tomography (CT) scans (including CT routine scan, CT-angiography, and CT-perfusion) at baseline, and neuroimaging reexamination (CT or MRI) within 24 to 48 h; 3) subjects who underwent EVT had the digital subtraction angiography (DSA) data; 4) they were followed-up at the three-month interval. The subjects met none of the exclusion criteria, as follows: 1) the mRS score > 2 before the AIS; 2) incomplete blood pressure data.

### Demographic and clinical data

Demographic data were recorded for each subjects, including age, sex, and body mass index (BMI). Past and current medical histories were also recorded. Particularly, in the current medical history, we recorded following items: 1) the onset-to-needle time (ONT) and door-to-needle time (DNT); 2) the National Institute of Health Stroke Scale (NIHSS) score at baseline, two-hour after therapy (2-h NIHSS), 24 h after therapy (24-h NIHSS), and 7 days after therapy (7-d NIHSS); and 3) incidence of hemorrhagic transformation and Alberta Stroke Program Early CT Score (ASPECTS).

### BP measurement

BP, including systolic BP (SBP) and diastolic BP (DBP), were measured at baseline and after reperfusion therapy. Specifically, we divided the timeline after stroke onset into four stages: baseline, 0–2 h after reperfusion therapy (hereinafter referred to as “2-h stage”), 2–24 h after reperfusion therapy (hereinafter referred to as “24-h stage), and 1–7 days after reperfusion therapy (hereinafter referred to as “7-d stage”). At baseline, the BP was measured once. In the 2-h stage, we measured the subject’s BP every 15 min. In the 24-h stage, we measured the subject’s BP once per hour. In the 7-d stage, we measured the subject’s BP once per day at 8: 00 AM. The baseline and 7-d stage BP were measured by an upper arm blood pressure monitor (Omron, HEM-7121). The 2-h stage and 24-h stage BP were measured by a patient monitor (Mindray, uMEC10). 

### Calculation of BPV

Four measurements, including SD, CV, ARV, and R, were applied to evaluate the variability of SBP and DBP during and after reperfusion therapy, respectively. Specifically, the SD measures the amount of the BP variation, which was calculated as the square root of the variance. The CV was defined as the SD divided by the mean of the BP. The ARV was the mean of the absolute differences of the consecutive measurements of BP, which is calculated by the equation below:

$$\mathrm{ARV}=\frac{1}{N-1}{\sum }_{k=1}^{n-1}\times \left|{BP}_{K+1}-\left.{BP}_{K}\right|\right.$$

where k ranges from 1 to N-1, and N is the number of the measurements of BP. The R is defined as the difference of the maximum and the minimum of the measured BP. The above four measurements were calculated for the 2-h stage, 24-h stage, and 7-d stage, respectively.

### Statistical analysis

Independent two-sample *t*-test and chi-square test were employed to compare quantitative and qualitative demographic and clinical data, respectively, between the good outcome and the poor outcome groups. The statistical significance was set at *p* < 0.05.

Independent two-sample *t*-test was used to compare the four BPV measurements between the good outcome and the poor outcome groups for each stage of the stroke. We used the ROC curve to evaluate the power of the measurements to differentiate the good outcome subjects from the poor outcome subjects. The area under the ROC (AUC) values were employed to compare the power of differentiation for the measurements. The optimal cutoff values of the measurements were then extracted. In addition, variables with significant differences between the two groups entered a multiple logistic regression analysis to identify the variables that were associated with poor outcome.

## Results

### Demographic and clinical characteristics

As shown in Table 1, compared with the poor outcome group, the good outcome was significantly younger (*t* = -2.65, *p* = 0.01), and exhibited significantly higher ASPECTS at baseline (*t* = 10.73, *p* < 0.01) and lower NIHSS scores at each stage of the stroke (baseline: *t* = -7.18, *p* < 0.01; 2-h after stroke: *t* = -7.25, *p* < 0.01; 24-h after stroke: *t* = -12.16, *p* < 0.01; 7-d after stroke: *t* = -16.46, *p* < 0.01).

**Table 1:** Demographic and clinical data between groups

	Poor outcome group (*n* = 62)	Good outcome group (*n* = 174)	*t* or *χ*2	*P* values
Female, n (%)	13 (21.0)	60 (34.5)	0.137	0.71
Age (years)	72.19 ± 12.49	67.32 ± 12.29	-2.65	0.01 ^a^
Body Mass Index	24.14 ± 3.48	25.07 ± 4.48	1.18	0.24
Smoking history, n (%)	7 (11.3)	48 (27.6)	6.79	0.01 ^a^
Hypertension, n (%)	44 (71.0)	107 (61.5)	1.78	0.18
Hyperglycemia, n (%)	11 (17.7)	49 (28.2)	2.62	0.11
Hyperlipidemia, n (%)	9 (14.5)	24 (13.8)	0.02	0.89
Coronary heart disease, n (%)	22 (35.5)	38 (21.8)	4.70	0.03 ^a^
Heart failure, n (%)	12 (19.4)	23 (13.2)	1.36	0.24
Atrial fibrillation, n (%)	16 (25.8)	21 (12.1)	6.53	0.01 ^a^
Stroke history, n (%)	27 (43.5)	16 (9.2)	6.26	0.04 ^a^
ONT (min)	191.23 ± 84.12	181.84 ± 67.22	-0.88	0.38
DNT (min)	58.44 ± 24.38	60.62 ± 32.6	0.44	0.66
Baseline NHISS	13.11 ± 5.90	7.59 ± 4.93	-7.183	< 0.001 ^a^
2-h NHISS	11.10 ± 6.35	5.41 ± 4.89	-7.25	< 0.001 ^a^
24-h NHISS	11.79 ± 3.97	3.97 ± 3.81	-12.16	< 0.001 ^a^
7-d NHISS	11.19 ± 5.62	2.37 ± 2.80	-16.46	< 0.001 ^a^
Hemorrhagic transformation, n (%)	10 (16.1)	15 (8.6)	2.72	0.10
ASPECTS	7.40 ± 1.08	8.98 ± 0.97	10.728	0.001^a^
Baseline SBP	153.47 ± 20.90	153.9 ± 22.17	0.14	0.89
Baseline DBP	85.66 ± 16.26	88.53 ± 16.36	1.19	0.24

Data are presented as mean ± stand deviation (SD)

*Abbreviations*: *n* number, *min* minute *ONT* onset-to-needle time, *DNT* door-to-needle time, *h* hour, *d* day, *NIHSS* National Institute of Health Stroke Scale, *ASPECTS* Acute Stroke Alberta Stroke Program Early CT score, *SBP* systolic blood pressure, *DBP* diastolic blood pressure

^a^indicates significant differences between groups

### Between-group differences in BPV

The between-group comparison of BPV is shown in Table 2. The two groups showed no significant difference in SBP or DBP at baseline. However, the good outcome group showed less BPV at each stage of stroke relative to the poor outcome group. Specifically, in the 24-h and 7-d stages, all of the four measurements, i.e., SD, CV, ARV, and R, were significant lower in the good outcome group compared with the poor outcome group. In the 2-h stage, the SD, CV, and ARV were significantly lower in the good outcome group compared with the poor outcome group.

**Table 2:** Measurements of blood pressure variability between groups

	Good outcome group (*n* = 62)	Poor outcome group (*n* = 174)	*t* or *χ*2	*P* values
**2-h SBP**				
SD	11.59 ± 4.63	9.22 ± 4.41	-3.58	< 0.001^a^
CV	13.05 ± 5.61	9.86 ± 5.58	-3.85	< 0.001 ^a^
ARV	11.15 ± 4.57	8.17 ± 4.00	-4.86	< 0.001 ^a^
R	19.68 ± 14.45	16.98 ± 13.10	1.35	0.177
**2-h DBP**				
SD	8.46 ± 3.90	7.30 ± 3.46	-2.19	0.03 ^a^
CV	10.25 ± 4.70	8.64 ± 4.63	-2.34	0.02 ^a^
ARV	8.82 ± 4.06	7.19 ± 3.52	-3.01	0.003 ^a^
R	26 ± 11.45	24.18 ± 11.68	1.22	0.23
**24-h SBP**				
SD	13.69 ± 3.93	11.33 ± 3.61	-4.32	< 0.001 ^a^
CV	14.51 ± 4.48	11.96 ± 3.71	-4.39	< 0.001 ^a^
ARV	11.27 ± 3.34	9.45 ± 2.74	-4.22	< 0.001 ^a^
R	60.32 ± 15.40	51.56 ± 16.55	-3.64	< 0.001 ^a^
**24-h DBP**				
SD	9.44 ± 2.36	8.73 ± 2.81	-1.79	0.08
CV	10.99 ± 3.24	9.91 ± 3.10	-2.32	0.02 ^a^
ARV	8.76 ± 2.60	7.79 ± 2.38	-2.69	0.008 ^a^
R	43.23 ± 11.21	39.78 ± 11.82	1.99	0.047 ^a^
**7-d SBP**				
SD	11.33 ± 5.07	9.27 ± 4.76	-2.80	0.01 ^a^
CV	14.30 ± 8.17	11.74 ± 6.91	-2.31	0.02 ^a^
ARV	13.76 ± 8.17	10.77 ± 6.02	-2.96	< 0.001 ^a^
R	63.52 ± 15.06	55.22 ± 17.37	3.34	0.001 ^a^
**7-d DBP**				
SD	7.95 ± 3.16	6.33 ± .54	-3.95	< 0.001 ^a^
CV	10.66 ± 5.51	8.30 ± 3.67	-3.67	< 0.001 ^a^
ARV	8.76 ± 2.60	7.79 ± 2.38	-2.69	0.01 ^a^
R	44.82 ± 11.01	41.21 ± 11.36	2.172	0.031 ^a^

Data are presented as mean ± stand deviation (SD). The 2-h stage and 24-h stage BP were measured by a patient monitor (Mindray, uMEC10). The 7-d stage BP were measured by an upper arm blood pressure monitor (Omron, HEM-7121)

*Abbreviations*: *n* number, *h* hour, *d* day, *SBP* systolic blood pressure, *DBP* diastolic blood pressure, *SD* standard deviation, *CV* coefficient of variation, *ARV* average real variability, *R* range

^a^indicates significant differences between groups

### Differentiation power of BPV measurements

**Fig. 1:**
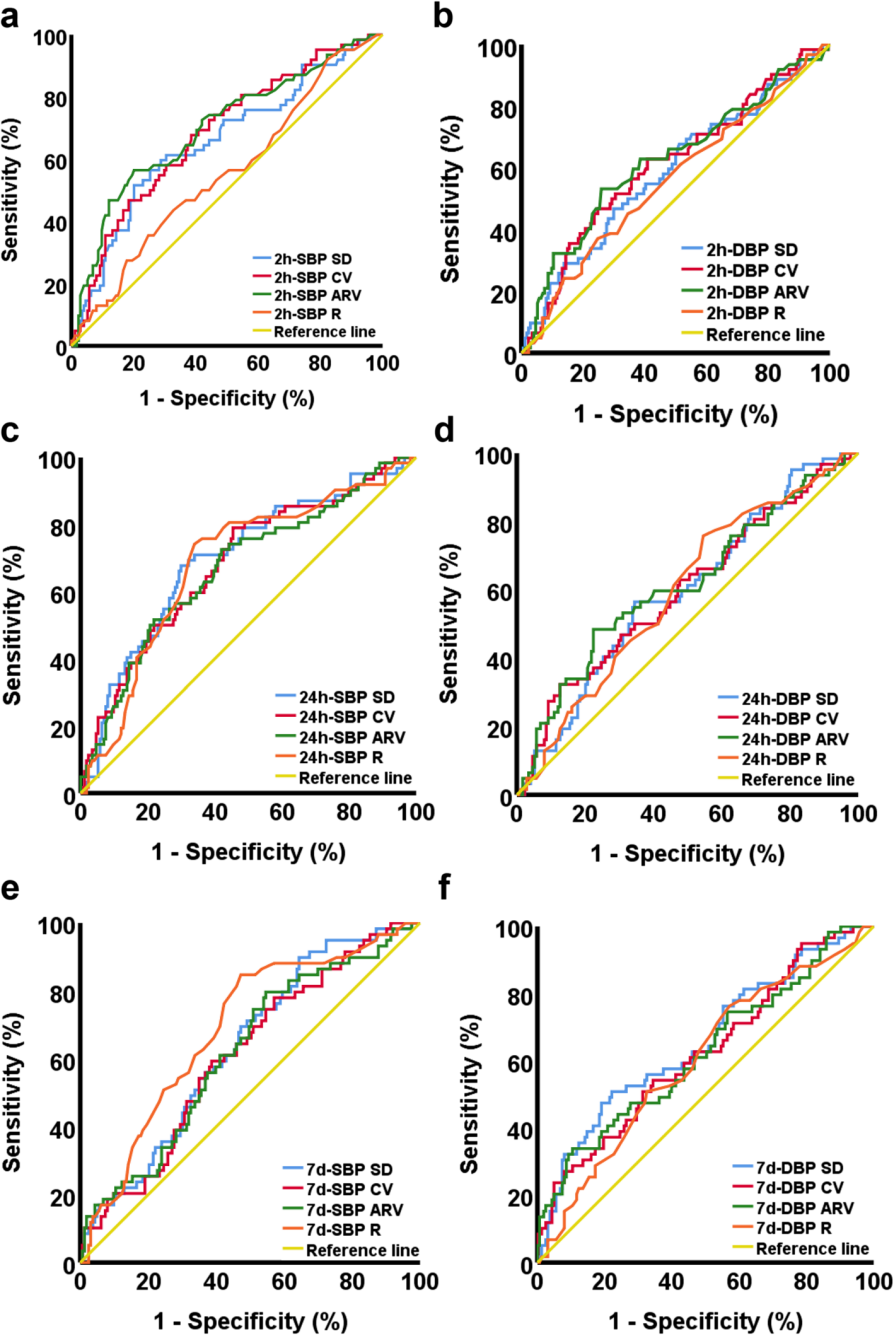
ROC curves of BPV measurements to differentiate good outcome and poor outcome subjects. ROC curves of systolic BPV at 2-h, 24-h, 7-day stages are shown in Figures **A**, **C**, **E**, respectively. ROC curves of diastolic BPV at 2-h, 24-h, 7-day stages are shown in Figures **B**, **D**, **F**, respectively. The 2-h stage and 24-h stage BP were measured by a patient monitor (Mindray, uMEC10). The 7-d stage BP were measured by an upper arm blood pressure monitor (Omron, HEM-7121). Abbreviations: ROC, receiver operating characteristic; BPV, blood pressure variability; h, hour; d, day

The ROC curve of each BPV measurement to differentiate good and poor outcome is shown in Fig. 1. Specifically, in the 2-h stage, the AUCs of SD, CV, and ARV in SBP were 0.657 (*p* = 0.001), 0.682 (*p* < 0.001), and 0.701 (*p* < 0.001), respectively; the AUCs of SD, CV, and ARV in DBP were 0.589 (*p* = 0.037), 0.611 (*p* = 0.01), and 0.624 (*p* = 0.004), respectively. In the 24-h stage, the AUCs of SD, CV, ARV, and R in SBP were 0.699 (*p* < 0.001), 0.684 (*p* < 0.001), 0.671 (*p* < 0.001), and 0.685 (*p* < 0.001), respectively; the AUCs of SD, CV, ARV, and R in DBP were 0.595 (*p* = 0.026), 0.598 (*p* = 0.023), 0.614 (*p* = 0.008), and 0.594 (*p* = 0.027), respectively. In the 7-d stage, the AUCs of SD, CV, ARV, and R in SBP were 0.634 (*p* = 0.002), 0.612 (*p* = 0.011), 0.619 (*p* = 0.007), and 0.688 (*p* < 0.001), respectively; the AUCs of SD, CV, ARV, and R in DBP were 0.656 (*p* < 0.001), 0.624 (*p* = 0.005), 0.624 (*p* = 0.005), and 0.601 (*p* = 0.021), respectively.

### Clinical variables associated with the outcome

As illustrated in Table 3, the multiple logistic regression analysis identified that the R of SBP in the 24-h stage higher than 54 mmHg (β = 2.28, *p* = 0.003), the ARV of DBP in the 24-h stage higher than 8.83 (β = 1.63, *p* = 0.021), and higher 7-d NIHSS score (β = 0.63, *p* < 0.001) were associated with poor outcome. By contrast, higher ASPECTS (β = -1.01, *p* = 0.001) was associated with good outcome.

**Table 3:** Multiple logistic regression analysis for variables associated with clinical outcome

Variables	β	Exp(β) / OR (95% CI)	*P* value
24-h SBP-R ≥ 54 mmHg	2.28	9.81 (2.20–43.83)	0.003
24-h DBP-ARV ≥ 8.83	1.63	5.11 (1.28 ~ 20.42	0.021
7-d NIHSS	0.63	1.88 (1.49–2.36)	< 0.001
ASPECTS	-1.01	0.36 (0.20–0.66)	0.001

*Abbreviations*: *h* hour, *d* day, *NIHSS* National Institute of Health Stroke Scale, *ASPECTS* Acute Stroke Alberta Stroke Program Early CT score, *SBP* systolic blood pressure, *DBP* diastolic blood pressure, *ARV* average real variability, *R* range

## Discussion

This study investigated the association of BPV after reperfusion therapy with clinical outcome at three months of stroke onset. It demonstrated that in all stages after reperfusion therapy (i.e., 2-h, 24-h, and 7-d stages), the good outcome group showed significant less BPV compared with the poor outcome group. The BPV measurements, especially in the 24-h stage, exhibit potentials to differentiate good outcome and poor outcome subjects. Among all demographic and clinic variables, greater ARV of DBP and greater R of SBP at 24-h stage, together with higher 7-d NIHSS score and lower ASPECTS, were associated with poor clinical outcome. In all, these results suggest that greater BPV during the first week after reperfusion therapy is a risk factor of poor clinical outcome in AIS.

The fundamental finding of this study is that in AIS patients treated with reperfusion therapy, those with no or minimum disability at three months exhibit remarkably less BPV during the first week after reperfusion therapy relative to those with slight to severe disability. Prevalent guidelines for early management of AIS recommend a clear absolute BP level that should be controlled for patients treated with reperfusion therapy [1, 3]. Besides the absolute BP level, emerging evidence demonstrates that BPV also is an important contributor to clinical outcome for these patients [13]. With respect to the patients treated with IVT, less BPV during the first 24 h of stroke onset, represented by successive variability [10, 14] or BPV parameters such as R, SD, and CV [9], is linked to better clinical outcome at three months. By contrast, greater SBP variability during the first 6 h after IVT was associated with hemorrhage transformation [11]. With respect to the patients treated with EVT, studies also demonstrate the associations of greater BPV with more severe functional disability and higher mortality rate at three months, in addition to the detrimental effect of absolute high SBP level on clinical outcome [15]. This study corroborates prevailing studies on the association of greater BPV with worse clinical outcome at three months (Table 4). It further enhances current understanding in which such a detrimental effect of greater BPV would extend to the first week of the stroke onset. Basically, the mechanism underlying such an association may be related to the impairment of autoregulation of cerebral blood flow after stroke onset. As the impairment emerges, the amount of cerebral blood flow largely depends on the BP level [16]. In this way, greater BPV would advance cerebral edema, aggravate cerebral ischemia reperfusion injury, and increase the risk of hemorrhagic transformation [2, 17]. Therefore, BP management should involve BPV and cover the first week after the stroke onset.

**Table 4:** Previous studies on the association between blood pressure variability and clinical outcome in acute ischemic stroke

Study	Subject numbers	Reperfusion therapy	Initial NIHSS^a^	Duration of BP Monitoring	BPV Parameters	Results
Delgado-Mederos et al. [8], 2008	80	IVT	15 (10–19)	The first 24 h	SD of the mean	BPV is associated with greater diffusion-weighted imaging lesion growth and worse clinical course
Endo et al. [9], 2013	527	IVT	12 (7–18)	The first 25 h	R, SD, CV, SV	SBP variability was positively associated with symptomatic intracerebral hemorrhage and death
Kellert et al. [10], 2017	28,976	IVT	11 (7–17)	From 2 to 24 h after onset	SV	SV was associated with poor functional outcome
Liu et al. [11], 2016	461	IVT	10 (5–16)	The first 24 h	SD, SV, SVrise, SVdrop, SVrisemax, SVdropmax	High SBP variability during the first 6 h was related with severe hemorrhagic transformation
Rasmussen, et al. [12], 2018	128	EVT	NA	During the EVT procedure	ΔMABP and ARV	No statistically significant association between BP related parameters during EVT and outcome
Bennett et al. [14], 2018	182	EVT (86% of the patients received IVT)	16 (10–22)	The first 24 h at minimum, 152 patients were measured through 120 h	SD, CV, SV	Increased BPV consistently predict worse neurologic outcomes
Maïer et al. [15], 2018	343	EVT(64% of the patients received IVT)	16 (11–20)	During the EVT procedure	CV of PP and SBP	PP variability during EVT is an independent predictor of worse clinical outcome in AIS
Kim et al. [17], 2019	211	EVT(54% of the patients received IVT)	16 (11–20)	The first 24 h	SD, CV, SV, R, TR	Time-related BP variability in the first 24 h was correlated with sICH

*Abbreviations*: *NIHSS* National Institute of Health Stroke Scale, *BP* blood pressure, *BPV* blood pressure variability, *IVT* intravenous thrombolysis, *h* hour, *SD* standard deviation, *CV* coefficient of variation, *R* range, *SV* successive variation, *SBP* systolic blood pressure, *EVT* endovascular thrombectomy, *MABP* mean arterial blood pressure, *ARV* average real variability, *PP* pulse pressure, *AIS* acute ischemic stroke, *TR* time rate, *sICH* symptomatic intracerebral hemorrhage

^a^NIHSS is expressed as median (interquartile range)

As the BPV measurements in all stages showed significant differences between the good outcome and the poor outcome groups, the ROC curve analysis was then used to examine the differentiation power of the BPV measurements among the stages. It appears that the systolic BPV measurements at 24-h stages exhibit relatively larger AUC (0.671 to 0.699) compared with other stages. The result suggests that BPV during the first 24 h after reperfusion therapy is of significance in influencing clinical outcome three months later. As discussed above, prevalent studies demonstrated the detrimental effects of greater BPV during the first 24 h after reperfusion therapy on clinical outcomes. In addition, an earlier study revealed that systolic BPV at 24 h stage served as an independent predictor of lesion growth appeared in diffusion weighted image and worse clinical outcome [8]. A recent study demonstrated that pulse pressure variability at 24-h stage independently correlated with clinical outcome at three months of the stroke onset. Every 5-mmHg increase in the pulse pressure variability was related to a 36% decrease in the likelihood of independent functional outcome and a 60% increase in the odds of mortality at three months of stroke onset [18]. Furthermore, a meta-analysis suggests that systolic BPV measurements during the first 24 h after EVT, including SD, CV, and successive variation, is indicative of clinical outcome at three months of stroke onset [19]. Therefore, maintaining less BPV at 24-h stage, relative to other stages, is particularly essential in promoting better clinical outcome in AIS.

**Fig. 2:**
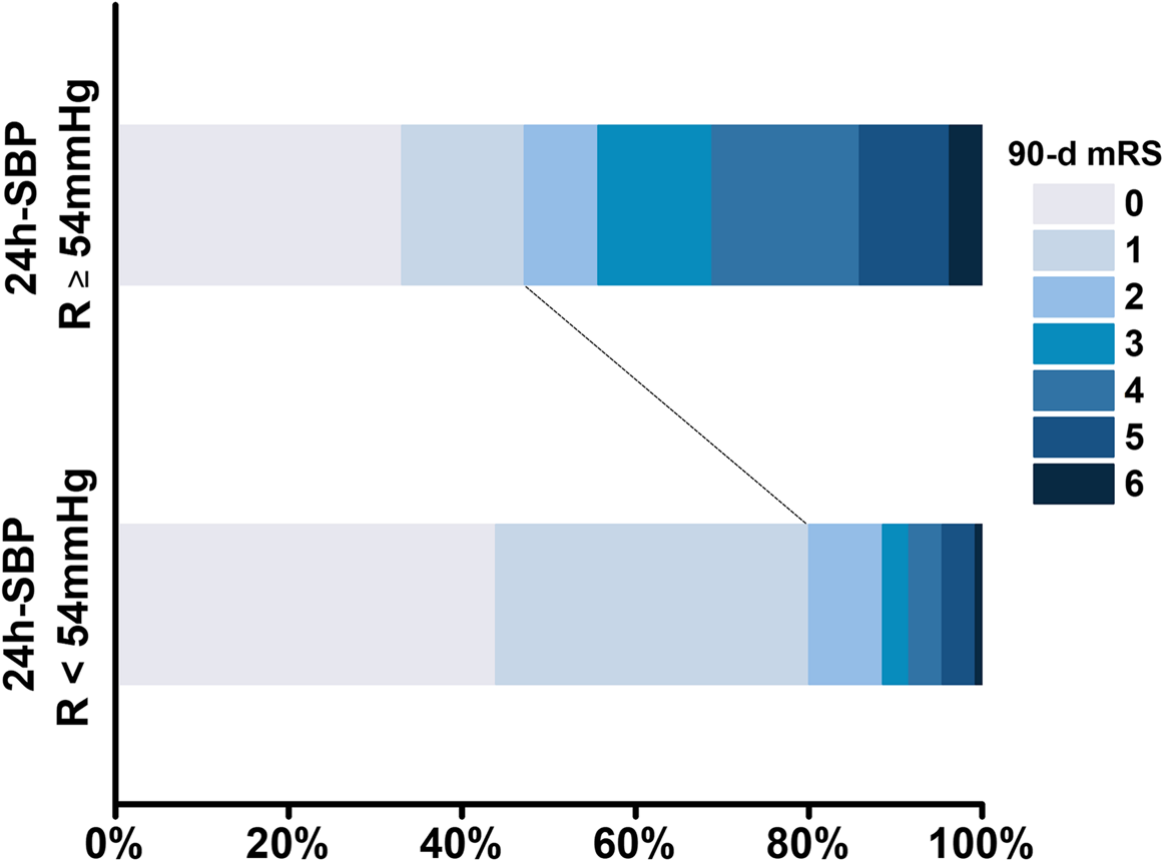
Distributions of 90-day mRS score by range of 24-h SBP cutoff at 54 mmHg. The proportion of good outcome (i.e., mRS score ≤ 2) was remarkably larger in the group of the R of SBP lower than 54 mmHg relative to the group of the R of SBP equal or higher than 54 mmHg. Abbreviations: mRS, Modified Rankin Scale; SBP, systolic blood pressure; R, ragne

The results from this study further broaden the association of BPV at the 24-h stage with clinical outcome, based on the cut-off value calculated by the ROC curve analysis. First, among all variables showing significant between-group differences, logistic regression analysis identified the R of SBP at the 24-h stage greater than 54 mmHg, the ARV of DBP at the 24-h stage greater than 8.83, together with greater 7-d NIHSS score and lower ASPECTS that were associated with clinical outcome at three months. Second, the distribution of 90-d mRS score by the R of SBP at the 24-h stage illustrates that the proportion of good outcome (i.e., mRS score was less than two) was remarkably larger in the group of the R of SBP lower than 54 mmHg relative to the group of the R of SBP equal or higher than 54 mmHg (Fig. 2). These two results not only highlight the contribution of BPV at the 24-h stage to the stroke outcome, but also indicate that the R of SBP could be used as a potential tool to control the BPV after reperfusion therapy. Currently, most of the studies qualitatively investigated the association of BPV and other factors with stroke outcome, but did not quantitatively indicate how to manage BPV for stroke patients after reperfusion therapy. This study examined the potential of the cut-off value of the BPV measurements in improving clinical outcome for the patients. The result suggests that maintaining the R of SBP less than 54 mmHg during the first 24 h after reperfusion therapy would benefit in advancing better prognosis for AIS patients treated with reperfusion therapy. In addition, given that keeping absolute BP level below a certain level is required during and after reperfusion therapy, timely reperfusion therapy may contribute to decrease the R of SBP, thus decreasing BPV and advancing better clinical outcome. Future studies are needed to test whether this hypothesis works and determine the ideal time to perform reperfusion therapy.

This study has following limitations. First, this is a retrospective study that examines associations between BPV measurements and stroke prognosis. Other factors, such as age, NIHSS score, and ASPECTS in this study, also showed significant between-group differences, which may emerge as confounding factors herein. A future study with a prospective design is essential to control the effect of these factors on the association. Second, the diagnostic value of the BPV measurements calculated by the ROC curve analysis is relatively low. This result suggest that other factors may also interact with BPV measurements to modulate prognosis. Additional studies are needed to validate current results and to further develop a framework involving BPV measures and other factors to predict AIS outcome in the early disease stage.

## Conclusions

This study demonstrates that good outcome of AIS treated with reperfusion therapy is associated with less BPV during the first week of the disease onset. BPV measurements, especially those during the first 24 h after reperfusion therapy, has the potential to differentiate good and poor outcome patients. The BPV measurements serves as an essential factor to modulate clinical outcome of the patients. The results underscore the role of BPV management in AIS treatment, and support the involvement of BPV management in future clinical guidelines on AIS.

## Data Availability

The datasets used and/or analysed during the current study are available from the corresponding author on reasonable request.

## Abbreviations

IVT: Intravenous thrombolysis
EVT: Endovascular thrombectomy;
AIS: Acute ischemic stroke
BP: Blood pressure;
BPV: Blood pressure variability
SD: Standard deviation
CV: Coefficient of variation
ARV: Average real variability
R: Range
mRS: Modified Rankin Scale
ROC: Receiver operating characteristic
CT: Computerized tomography
DSA: Digital subtraction angiography
BMI: Body mass index
ONT: Onset-to-needle time
DNT: Door-to-needle time
NIHSS: National Institute of Health Stroke Scale
ASPECTS: Alberta Stroke Program Early CT Score
SBP: Systolic blood pressure
DBP: Diastolic blood pressure
AUC: Area under the ROC
